# Supplementary material for: Random time-shift approximation enables hierarchical Bayesian inference of mechanistic within-host viral dynamics models on large datasets
Source: PLoS Comput Biol. 2025 Dec 4;21(12):e1013775. doi: 10.1371/journal.pcbi.1013775 (PMC12688156; doi:10.1371/journal.pcbi.1013775)
Supplement: S1 text — This text includes the derivation of the deterministic approximation and the key branching process results underlying the time-shift distributions. We provide a full derivation of the Laplace approximation and compare it with the exact calculation using profile likelihoods (Fig 1 in S1 text). Further details are given on the model and inference method, including the MCMC routine, as well as the construction of the neural network used for amortised optimisation. (PDF) [file pcbi.1013775.s001.pdf]

Supporting Information: Random time-shift approximation enables hierarchical Bayesian inference of mechanistic within-host viral dynamics models on large datasets

Dylan J. Morris, Lauren Kennedy, Andrew J. Black

S1 text: Supporting information

1 Deterministic approximation

Let $\boldsymbol{x}(t) = K^{-1}\boldsymbol{X}(t)$ denote the density process of the CTMC $\{\boldsymbol{X}(t), t \geq t_0\}$. Then reading down column 2 of Table 1 in the main text, and provided $\boldsymbol{a} + \boldsymbol{l} \in \mathcal{S}$ where $\mathcal{S}$ is the state space of the CTMC, $\boldsymbol{a}$ is the current state and $\boldsymbol{l}$ represents the jumps of the process, then the (positive) transition rates can be expressed in terms of the following functions

$$r^{(K)}(\boldsymbol{a}, \boldsymbol{a}+\boldsymbol{l}) = \begin{cases} K\alpha sv, & \text{if } \boldsymbol{\ell} = (-1, 1, 0, 0), \\ Kke, & \text{if } \boldsymbol{\ell} = (0, -1, 1, 0), \\ K\delta i, & \text{if } \boldsymbol{\ell} = (0, 0, -1, 0), \\ K\rho i, & \text{if } \boldsymbol{\ell} = (0, 0, 0, 1), \\ Kcv, & \text{if } \boldsymbol{\ell} = (0, 0, 0, -1). \end{cases} \tag{1}$$

Since the rates can be expressed in this form (referred to as "density dependent"), by Theorem 3.1 of [1], as $K \to \infty$ the process $\boldsymbol{x}(t)$ converges to a deterministic process $\boldsymbol{x}_d(t)$ uniformly in probability over finite time intervals provided $\boldsymbol{x}_d(0) = K^{-1}\boldsymbol{X}(0)$. Letting

$$\boldsymbol{H}(\boldsymbol{x}_d(t)) = \sum_{\boldsymbol{l}} \boldsymbol{l} r(\boldsymbol{a}, \boldsymbol{a}+\boldsymbol{l}),$$

where $r(\boldsymbol{a}, \boldsymbol{a}+\boldsymbol{l}) = K^{-1}r^{(K)}(\boldsymbol{a}, \boldsymbol{a}+\boldsymbol{l})$, then the system of ODEs governing the deterministic approximation is given by

$$\frac{\mathrm{d}\boldsymbol{x}_d(t)}{\mathrm{d}t} = \boldsymbol{H}(\boldsymbol{x}_d(t)). \tag{2}$$

Let $s(t) = S(t)/K$, $e(t) = E(t)/K$, $i(t) = I(t)/K$ and $v(t) = V(t)/K$ denote the densities of the

cell and virus populations of each type, then in the limit as $K \to \infty$ these evolve via a set of coupled ODEs [1] (where the d subscript means these are deterministic)

$$\begin{aligned} \frac{\mathrm{d}s_d(t)}{\mathrm{d}t} &= -\alpha s_d(t)v(t), \\ \frac{\mathrm{d}e_d(t)}{\mathrm{d}t} &= \alpha s_d(t)v_d(t) - ke_d(t), \\ \frac{\mathrm{d}i_d(t)}{\mathrm{d}t} &= ke_d(t) - \delta i_d(t), \\ \frac{\mathrm{d}v_d(t)}{\mathrm{d}t} &= \rho i_d(t) - cv_d(t). \end{aligned} \tag{3}$$

We can multiply Eqs (3) through by the volume K as it is large and fixed to obtain the system of ODEs that govern the approximation for the mean dynamics (Eq (2) in the main text) [2,3].

2 Branching process results

Considering the early stages of infection within a host, the number of target cells, S, is approximately constant, i.e. $S \approx S_0$ and hence the rates in Table 1 of the main text are linear. The BPA then tracks the evolution of the state vector $\boldsymbol{X}_b(t) = (E, I, V)$ and the change in event and corresponding rates are given in Table 1.

$\Delta\boldsymbol{X}$	Rate
$(1,0,0)$	$\beta S_0 V$
$(-1,1,0)$	kE
$(0,-1,0)$	δI
$(0,0,1)$	ρI
$(0,0,-1)$	cV

Table 1: Change in state and rates for the BPA to the TCL model.

To apply the method in [4], we must identify the constant terms from the progeny generating functions which are defined as

$$f_i(\boldsymbol{s}) = \sum_{\boldsymbol{k}} p_i(\boldsymbol{k}) \prod_{j=1}^{m} s_j^{k_j}, \quad \boldsymbol{s} \in [0,1]^m$$

where $p_i(\boldsymbol{k})$ is the probability of an individual of type i having $\boldsymbol{k} = (k_1, \ldots, k_m)$ offspring of each

type [4]. Let $\beta^\star = \beta S_0$, then for the TCL model

$$
\begin{aligned}
f_1(\boldsymbol{s}) &= \frac{k}{k} s_2 = s_2 \\
f_2(\boldsymbol{s}) &= \frac{\delta + \rho s_2 s_3}{\delta + \rho} \\
f_3(\boldsymbol{s}) &= \frac{c + \beta^\star s_1 s_3}{c + \beta^\star}.
\end{aligned}
$$

The non-zero constants (see [4] for details) needed for the time-shift computations are

$$\alpha_{12} = k, \quad \beta_{223} = \rho, \quad \beta_{313} = \beta^\star.$$

3 Laplace approximation derivation

We evaluated two approaches: (1) constructing a Laplace approximation for the path-likelihood alone and then numerically integrating, or (2) constructing a Laplace approximation for the product of the path-likelihood and the time-shift distribution, and deriving a closed-form expression. The approximation of the path-likelihood alone was initially considered the more appropriate approach, as the path-likelihood is the product of normal PDFs (and few CDF values), and therefore, it is approximately a scaled Normal distribution (note that the scaling means this is a function, not a density). In contrast, the time-shift distribution is skewed (see Fig 1 in the main text). However, in our testing, we found that these two approaches produced comparable results, with the Laplace approximation of the product of the path-likelihood and time-shift distribution reducing the evaluation time of the likelihood by approximately 30%.

We begin the construction of the Laplace approximation by noting that the data, $\boldsymbol{y}_i$, VL model parameters, $\boldsymbol{\theta}_i$, and κ are assumed to be known and fixed. Define the function

$$g(\tau) = \log f(\boldsymbol{y}_i \mid \boldsymbol{z}_i(\tau), \boldsymbol{\theta}_i, \kappa) + \log f(\tau \mid \boldsymbol{\theta}_i).$$

Suppose there is a global maximum

$$\tau_0 = \arg\max_{\tau} g(\tau).$$

Taylor expanding $g(\tau)$ about τ_0 and noting that $h'(\tau_0) = g'(\tau_0) = 0$ (since τ_0 is the maximum), we get

$$g(\tau) \approx g(\tau_0) + \frac{1}{2} g''(\tau_0)(\tau - \tau_0)^2 = g(\tau_0) - \frac{1}{2G(\tau_0)^2}(\tau - \tau_0)^2.$$

where

$$G(\tau) = \sqrt{-\frac{1}{g''(\tau)}},$$

noting that $g(\tau)$ is assumed to be concave (implicit in applying the Laplace approximation), which implies $g''(\tau) < 0$ around τ_0, and hence $G(\tau) > 0$. Substituting this into Eq (7) in the main text gives the Laplace approximation

$$f(\boldsymbol{y}_i \,|\, \boldsymbol{\theta}_i, \kappa) \approx f(\boldsymbol{y}_i \mid \boldsymbol{z}_i(\tau_0), \boldsymbol{\theta}_i, \kappa) f(\tau_0 \mid \boldsymbol{\theta}_i) \int \exp\left(-\frac{(\tau - \tau_0)^2}{2G(\tau_0)^2}\right) \mathrm{d}\tau.$$

Since the interior of the integrand is a Normal kernel, a closed-form approximation is given by

$$f(\boldsymbol{y}_i \,|\, \boldsymbol{\theta}_i, \kappa) \approx \sqrt{2\pi} f(\boldsymbol{y}_i \mid \boldsymbol{z}_i(\tau_0), \boldsymbol{\theta}_i, \kappa) f(\tau_0 \mid \boldsymbol{\theta}_i) G(\tau_0), \tag{4}$$

or, more compactly letting $h(\tau) = e^{g(\tau)}$,

$$f(\boldsymbol{y}_i \,|\, \boldsymbol{\theta}_i, \kappa) \approx \frac{\sqrt{2\pi} h(\tau_0)}{\sqrt{-g''(\tau_0)}}, \tag{5}$$

which is Eq (10) in the main text. The mean of the Gaussian, τ_0, can be estimated using numerical optimisation. The scale term $G(\tau_0) = (-g''(\tau_0))^{-1/2}$ has a more complicated form but can be computed through either numerical differentiation or automatic differentiation. In this work, all our code is written in the Julia programming language [5], and the package ForwardDiff.jl [6] enables efficient computation of exact derivatives through forward mode automatic differentiation for such codes.

4 Comparison of our Laplace approximation versus the exact calculation

Fig 1 shows the profile likelihoods for a given parameter set (for a random individual). These fix all but the parameter of interest and allow that to vary over some interval. The orange dots show our approximation and we can see strong agreement between the approximation and the "exact" likelihood. The maximum (absolute) error computed over the points shown in the plot is $< 1 \times 10^{-7}$.

5 Model

Relationships between the components in the model are depicted in the plate diagram shown in Fig 2 where the direction of the arrows dictates dependencies between parameters, hyper-parameters and data.

The joint posterior distribution of the model parameters and the shared parameters is given by

$$f(\boldsymbol{\Theta}, \boldsymbol{\phi}, \kappa \,|\, \mathcal{D}) \propto f(\mathcal{D} \,|\, \boldsymbol{\Theta}, \kappa) f(\boldsymbol{\Theta}|\boldsymbol{\phi}) f(\boldsymbol{\phi}) f(\kappa), \tag{6}$$

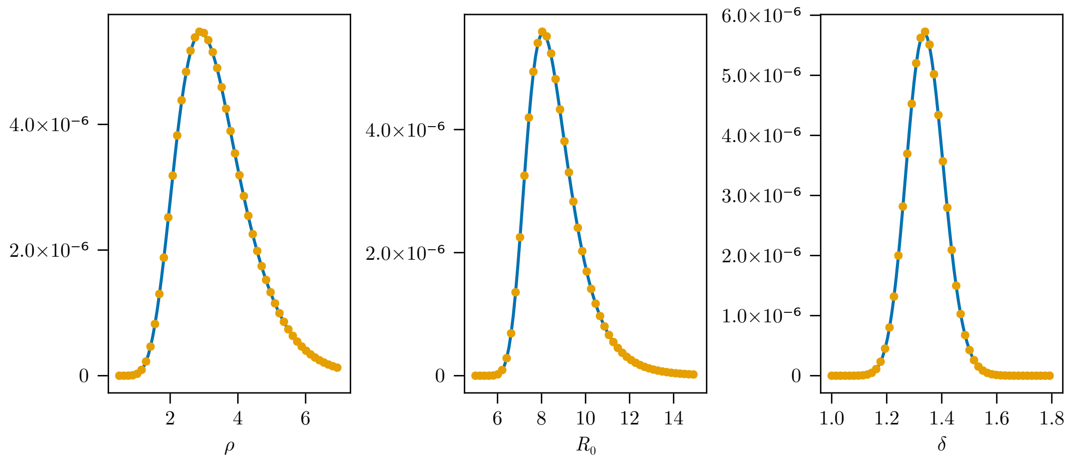

Figure 1: **Profile likelihoods for the exact method and the Laplace approximation.** Profile likelihoods for the two methods: exact given by the solid blue line, Laplace approximation in orange points.

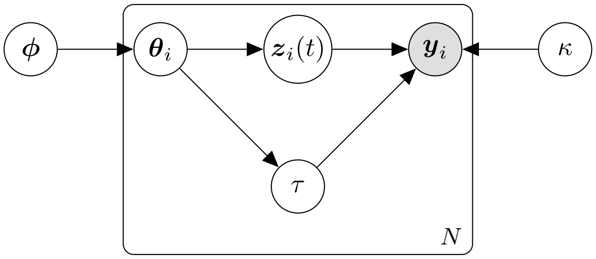

Figure 2: **Plate diagram of the relationship between components in the model.** The plate indicates individual level variables that are indexed by the number of individuals, N. The parameters outside the plate, $\boldsymbol{\phi}$ and κ, are shared across all individuals.

where $f(\mathcal{D} \,|\, \boldsymbol{\Theta}, \kappa)$ is the joint parameter-likelihood, and $f(\boldsymbol{\Theta}|\boldsymbol{\phi})$, $f(\boldsymbol{\phi})$, and $f(\kappa)$ are prior distributions that are detailed in Section 2.6. Since the individuals are conditionally independent given their unique parameters, $\boldsymbol{\theta}_i$, we can factorise the joint-likelihood as

$$f(\mathcal{D} \,|\, \boldsymbol{\Theta}, \kappa) = \prod_{i=1}^{N} f(\boldsymbol{y}_i \,|\, \boldsymbol{\theta}_i, \kappa), \tag{7}$$

where $f(\boldsymbol{y}_i \,|\, \boldsymbol{\theta}_i, \kappa)$ is the *marginal likelihood* function for individual i, as it is obtained by marginalising over the random time-shifts (see Section 2.5 for details).

5.1 Inference

The form of the joint posterior (Eq (5) in the main text) suggests a Gibbs sampler that draws from the conditional distributions for each of the individuals ($i = 1, \ldots, N$) and the shared parameters

$(\boldsymbol{\phi}, \kappa)$. However, sampling directly from the conditionals is not possible as the distributions cannot be normalised—or recognised as standard distributions—so we use a Metropolis-Hastings step within the Gibbs sampler. The conditional distributions for the individual parameters, noting that $\boldsymbol{\theta}_i$ are independent of $\boldsymbol{\Theta}_{-i} = \{\boldsymbol{\theta}_1, \ldots, \boldsymbol{\theta}_{i-1}, \boldsymbol{\theta}_{i+1}, \ldots, \boldsymbol{\theta}_N\}$ given $\boldsymbol{\phi}$, are given by

$$f(\boldsymbol{\theta}_i \,|\, \boldsymbol{\phi}, \kappa, \mathcal{D}) \propto f(\boldsymbol{y}_i \,|\, \boldsymbol{\theta}_i, \kappa) f(\boldsymbol{\theta}_i \,|\, \boldsymbol{\phi}).$$

The conditional distribution for κ and ϕ is

$$f(\boldsymbol{\phi}, \kappa \,|\, \boldsymbol{\Theta}, \mathcal{D}) \propto f(\kappa) f(\boldsymbol{\phi}) \prod_{i=1}^{N} f(\boldsymbol{y}_i \,|\, \boldsymbol{\theta}_i, \kappa) f(\boldsymbol{\theta}_i \,|\, \boldsymbol{\phi}). \tag{8}$$

Let the current draw be $\{\boldsymbol{\Theta}^{(k)}, \boldsymbol{\phi}^{(k)}, \kappa^{(k)}\}$ and we use the notation $\boldsymbol{\Theta}_{a:b} = \{\boldsymbol{\theta}_a, \ldots, \boldsymbol{\theta}_b\}$ for integers a and b ($b > a$). Define the following collections

$$\begin{aligned} \psi_i^{(k+1)} &= \left\{\boldsymbol{\Theta}_{1:i}^{(k+1)}, \boldsymbol{\Theta}_{i+1:N}^{(k)}\right\}, \quad i = 1, \ldots, N-1, \\ \psi_N^{(k+1)} &= \left\{\boldsymbol{\Theta}_{1:N}^{(k+1)}\right\}. \end{aligned}$$

We choose a relatively simple multivariate Gaussian proposal distributions centered on the current values of the parameters being drawn, with $N+1$ separate covariance matrices per step of the method, i.e.

$$\begin{aligned} \boldsymbol{\theta}_i' | \boldsymbol{\theta}_i^{(k)} &\sim \mathcal{N}(\boldsymbol{\theta}_i^{(k)}, \boldsymbol{\Sigma}_i), \quad i = 1, \ldots, N, \\ (\boldsymbol{\phi}', \kappa') | (\boldsymbol{\phi}^{(k)}, \kappa^{(k)}) &\sim \mathcal{N}((\boldsymbol{\phi}^{(k)}, \kappa^{(k)}), \boldsymbol{\Sigma}_{N+1}), \end{aligned}$$

Blocking the proposals in this way enables the subject specific proposals as well as the proposal for the shared parameters, κ and $\boldsymbol{\phi}$, to be tuned separately. The covariance matrices are tuned by performing a sufficiently large pilot run. Denote the PDF of the proposal distributions by $Q(\cdot \,|\, \cdot)$, where the arguments specify the particular proposal distribution being used. The acceptance probabilities for individual parameters ($i = 1, \ldots, N$) are given by

$$\alpha_{1,i} = \min \left\{ \frac{f(\boldsymbol{\theta}_i' \,|\, \psi_{i-1}^{(k+1)}, \boldsymbol{\phi}^{(k)}, \kappa^{(k)}, \mathcal{D})}{f(\boldsymbol{\theta}_i^{(k)} \,|\, \psi_{i-1}^{(k+1)}, \boldsymbol{\phi}^{(k)}, \kappa^{(k)}, \mathcal{D})} \times \frac{Q(\boldsymbol{\theta}_i^{(k)} \,|\, \boldsymbol{\theta}_i')}{Q(\boldsymbol{\theta}_i' \,|\, \boldsymbol{\theta}_i^{(k)})}, 1 \right\}, \tag{9}$$

Similarly, the acceptance probability for the shared parameters is

$$\alpha_2 = \min \left\{ \frac{f(\boldsymbol{\phi}', \kappa' \,|\, \psi_N^{(k+1)}, \mathcal{D})}{f(\boldsymbol{\phi}^{(k)}, \kappa^{(k)} \,|\, \psi_N^{(k+1)}, \mathcal{D})} \times \frac{Q(\boldsymbol{\phi}^{(k)}, \kappa^{(k)} \,|\, \boldsymbol{\phi}', \kappa')}{Q(\boldsymbol{\phi}', \kappa' \,|\, \boldsymbol{\phi}^{(k)}, \kappa^{(k)})}, 1 \right\}. \tag{10}$$

Note that the proposal distributions are symmetric and so the ratio of the proposal distributions in Eq (9) and Eq (10) are equal to 1. Algorithm 1 outlines a single step of the Metropolis-within-Gibbs

sampler.

Algorithm 1 **Single step of the Metropolis-within-Gibbs algorithm**

Inputs:
- Current draw $(\mathbf{\Theta}^{(k)}, \boldsymbol{\phi}^{(k)}, \kappa^{(k)})$;
- Conditional distributions; $f(\boldsymbol{\theta}_i \,|\, \boldsymbol{\phi}, \kappa, \mathcal{D})$, $f(\boldsymbol{\phi}, \kappa \,|\, \mathbf{\Theta}, \mathcal{D})$;
- Transition kernels; $Q(\boldsymbol{\theta}_i' \,|\, \boldsymbol{\theta}_i)$ for $i = 1, \ldots, N$, $Q(\boldsymbol{\phi}', \kappa' \,|\, \boldsymbol{\phi}, \kappa)$

1: **for** $i = 1, \ldots, N$ **do**
2:     Propose individual parameters $\boldsymbol{\theta}_i' \sim Q(\boldsymbol{\theta}_i' \,|\, \boldsymbol{\theta}_i^{(k)})$.
3:     Compute $\alpha_{1,i}$ (Eq (9)) and accept proposal with probability $\alpha_{1,i}$.
4: **end for**
5: Propose shared parameters $\boldsymbol{\phi}', \kappa' \sim Q(\boldsymbol{\phi}', \kappa' \,|\, \boldsymbol{\phi}^{(k)}, \kappa^{(k)})$.
6: Compute α_2 (Eq (10)) and accept proposal with probability α_2.

5.1.1 Parallelisation of the inference method

The structure of the inference algorithm and the form of the conditional distributions allow for straightforward parallelisation. The for loop (lines 1-4 of Algorithm 1) samples the VL model parameters $\boldsymbol{\theta}_i$ independently of the other individuals given the shared parameters. This means that the sampling of the individual parameters can be parallelised across the individuals. Additionally, parallelisation can also be used when computing the acceptance probability in line 6 as the likelihood contributions from each individual in Eq (8) (the $\mathcal{L}_i(\boldsymbol{\theta}_i, \kappa)$ terms inside the product) can be computed in parallel conditional on a draw of the hyper-parameters $\boldsymbol{\phi}$.

5.1.2 NCP

We use a non-centred parameterisation (NCP) for the individual-level parameters to enhance posterior sampling efficiency [7]. Specifically, the individual-specific parameters R_{0_i}, δ_i, and ρ_i are modelled as

$$R_{0_i} = \mu_{R_0} + \sigma_{R_0} z_{R_{0_i}}, \quad \delta_i = \mu_\delta + \sigma_\delta z_{\delta_i}, \quad \rho_i = \mu_\rho + \sigma_\rho z_{\rho_i}$$

where $z_{R_{0_i}}, z_{\delta_i}, z_{\rho_i} \sim \mathcal{N}(0,1)$. This parameterisation facilitated more efficient posterior sampling.

6 Neural network architecture and details

One particular challenge with using the time-shift approach in a statistical inference framework is that computing the parameters of the distribution is itself a computationally expensive task. Each call of the likelihood requires optimisation to be carried out to determine the parameters of the time-shift distribution [4]. To overcome this performance bottleneck, we utilise a neural network (NN) to learn the parameters of the time-shift distribution, (a, b, p), given VL model

parameters, $\boldsymbol{\theta} = (R_0, k, \delta, \rho, c)$. Note the removal of t_0 from the VL model parameters as the time-shift distribution does not depend on the infection time. This process is referred to as amortised optimisation [8–10] and is a common technique in machine learning to improve the efficiency of computationally complex algorithms such as Markov chain Monte Carlo [10].

Let $\boldsymbol{\zeta}_i = (a_i, b_i, p_i)$ denote the parameters of the time-shift distribution for some model parameters $\boldsymbol{\theta}_i$. We generate our dataset by sampling VL model parameters from a hyper-cube that encompasses our model priors. Our previous work [4] is used to determine the time-shift distribution parameters to give the paired data, $\{(\boldsymbol{\theta}_i, \boldsymbol{\zeta}_i)\}_{i=1}^{n}$. The time-shift distributions are checked for validity purposes since sampling on the hyper-cube does not necessarily produce feasible parameter combinations. The goal here is to learn a function $g : \mathbb{R}^5 \mapsto \mathbb{R}^3$ given by $g(\boldsymbol{\theta}_i) = \boldsymbol{\zeta}_i$ to a high degree of accuracy. The learned function will approximate the mapping from the VL model parameters to the time-shift parameters without the need to perform complex numerical routines. Replacing the numerical routine with this learned function makes the runtime to find the parameters approximately 200× faster.

When generating the training data, we sample parameters on a hyper-cube capturing the range of the independent priors used in the inference. In other words, the we sample the parameters as follows

$$
\begin{aligned}
R_0 &\sim U(1, 35) \\
\delta &\sim U(0.01, 10) \\
\rho &\sim U(0.01, 10).
\end{aligned}
$$

Instead of naively sampling parameters, we check that the growth rate, $\lambda > 0$, and that 95% of the density for τ is between $[-7, 7]$. The first condition ensures that there is a non-zero probability of the infection going non-extinct before reaching the exponential growth phase. The second condition ensures that long time-shifts, typically about a half of the total viral infection time-span (in our case), are considered un-biological and that time-shifts lying outside range likely suggests an incorrect model specification or poor prior choices. One key detail from an implementation perspective is that we also standardise the inputs (model parameters) and the outputs (time-shift parameters) using standard scaling (i.e. subtract the sample mean for each parameter and divide by the sample variance). In the context of training NN's, this standardisation has the effect of drastically improving the performance and convergence behaviour of optimisers like Adam. We utilise a simple NN with the following architecture:

1. Input layer: 3 neurons (the free parameters of the VL model) with ReLU activation
2. Hidden layer: 64 neurons with ReLU activation
3. Output layer: 3 neurons (the parameters of the time-shift distribution) with soft-plus activation

The input and hidden layer both use the rectified linear unit (ReLU) activation functions. The first of which acts to standardise the input data and then in the output layer we use the soft-plus activation function to ensure non-negativity in the GG parameters. We consider mean-squared-error (MSE) as the loss function to be optimised as this performed well in testing and all the output parameters for the surrogate distribution are on the same scale. Let

$$\mathcal{A} = \left\{(\boldsymbol{\theta}_i, \boldsymbol{\zeta}_i)\right\}_{i=1}^{n_{\text{train}}} \quad \text{and} \quad \mathcal{B} = \left\{(\boldsymbol{\theta}_i, \boldsymbol{\zeta}_i)\right\}_{i=1}^{n_{\text{test}}}$$

denote the training and validation sets respectively. Let $\mathbb{M}_\Psi(\boldsymbol{\theta})$ denote the current NN model—where we have collected the NN model parameters into Ψ—evaluated at mechanistic model parameters $\boldsymbol{\theta}$ which returns the surrogate distribution parameters. Define $\mathbb{M}(\boldsymbol{\theta})_j$ as the jth element of this (i.e. the estimated surrogate distribution parameter using the NN), then the loss is given by

$$L(\Psi) = \sum_{i=1}^{n_{\text{train}}} \sum_{j=1}^{3} \left(\mathbb{M}_\Psi(\boldsymbol{\theta}_{\boldsymbol{i}})_j - \zeta_j\right)^2. \tag{11}$$

We use the ADAM optimiser with defaults as provided by the Julia package Flux.jl. Additionally to improve the generalisability of the model, we perform early stopping. Early stopping uses the validation set and we stop training the model if there is no improvement in the loss (calculated in the same way as the loss in Eq (11)) on the validation set after 30 epochs. The model is completely implemented and trained in Julia on a CPU (to simplify dissemination of codes) using the Flux.jl package.

7 Simulation study and validation metrics

The simulation study was conducted primarily to evaluate the performance of the proposed inference method on datasets of substantial size. To validate the method, we used several scoring rules, specifically, *coverage*, *interval width*, and *relative bias*.

Consider a scalar population parameter θ used to generate M simulated datasets, $\{\mathcal{D}_i\}_{i=1}^M$. After fitting the model to each dataset, we obtain a posterior distribution for θ. Let

$$Q^{(i)} = \left(q_{0.025}^{(i)}, q_{0.975}^{(i)}\right)$$

denote the 95% equal-tailed credible interval for θ from dataset $\mathcal{D}_i$. The coverage, C, is defined as

$$C = \frac{1}{M} \sum_{i=1}^{M} \mathbb{I}_{\{\theta \in Q^{(i)}\}},$$

where $\mathbb{I}(\cdot)$ is the indicator function that equals 1 if the true parameter value θ lies within the interval $Q^{(i)}$, and 0 otherwise. The coverage is simply the number of 95% intervals that contain the true

parameter θ. The average interval width, W, is given by

$$W = \frac{1}{M} \sum_{i=1}^{M} \left(q_{0.975}^{(i)} - q_{0.025}^{(i)} \right),$$

providing a measure of the average uncertainty in the posterior estimates. Finally, the relative bias, B, is defined as

$$B = \frac{1}{M} \sum_{i=1}^{M} \frac{\hat{\theta}^{(i)} - \theta}{\theta},$$

where $\hat{\theta}^{(i)}$ is the posterior mean estimate of θ for dataset $\mathcal{D}_i$.

Each of the reported metrics (coverage, interval width, and relative bias) was summarised across 200 independently simulated datasets.

References

1. Kurtz TG. Solutions of Ordinary Differential Equations as Limits of Pure Jump Markov Processes. Journal of Applied Probability. 1970;7:49–58. doi:10.2307/3212147.

2. Zitzmann C, Ke R, Ribeiro RM, Perelson AS. How Robust Are Estimates of Key Parameters in Standard Viral Dynamic Models? PLOS Computational Biology. 2024;20:e1011437. doi:10.1371/journal.pcbi.1011437.

3. Baccam P, Beauchemin C, Macken CA, Hayden FG, Perelson AS. Kinetics of Influenza A Virus Infection in Humans. Journal of Virology. 2006;80:7590–7599. doi:10.1128/JVI.01623-05.

4. Morris D, Maclean J, Black AJ. Computation of Random Time-Shift Distributions for Stochastic Population Models. Journal of Mathematical Biology. 2024;89:33. doi:10.1007/s00285-024-02132-6.

5. Bezanson J, Edelman A, Karpinski S, Shah VB. Julia: A Fresh Approach to Numerical Computing. SIAM Review. 2017;59:65–98. doi:10.1137/141000671.

6. Revels J, Lubin M, Papamarkou T. Forward-Mode Automatic Differentiation in Julia; 2016.

7. Gelman A. Bayesian Data Analysis. City: CRC Press; 2013.

8. Amos B. Tutorial on Amortized Optimization. Foundations and Trends in Machine Learning. 2023;16:592–732. doi:10.1561/2200000102.

9. Marino J, Cvitkovic M, Yue Y. A General Method for Amortizing Variational Filtering. In: Advances in Neural Information Processing Systems. vol. 31. Curran Associates, Inc.; 2018.

10. Gershman S, Goodman N. Amortized Inference in Probabilistic Reasoning. Proceedings of the Annual Meeting of the Cognitive Science Society. 2014;36.
